# The Book World of Medicine and Science

**Published:** 1896-05-09

**Authors:** 


### THE BOOK WORLD OF MEDICINE AND SCIENCE.

Diagnosis and Treatment of Diseases of the Rectum, Anus, and Contiguous Textures. By S. G. Gant, M.D. With two chapters by Herbert William Allingham, F.R.C.S.Eng. (Philadelphia: The F. A. Davis Company. 1896. Pp. 400. Illustrated with wood engravings, and chromo-lithographs.)

This is a thoroughly practical work, and does not dive down so deeply into purely special questions as to detract from its interest to the general practitioner. Nevertheless, accuracy is by no means sacrificed to simplicity, and we can confidently recommend it as a book the study of which will be of much service to many, more especially to those who have not had the opportunity of watching recent advances in this branch of surgery. The descriptions of the operations are very graphic, and the illustrations add greatly to the readiness with which the author's meaning can be grasped by the reader. In the treatment of piles, Whitehead's operation is condemned as inferior to both ligature and cautery. The clamp and cautery operation is the one recommended, and for its more effectual performance the author has devised a clamp which certainly appears to obviate many of the disadvantages which have been found in several of the clamps hitherto in use. In speaking of the treatment of fistula-in-ano by division, emphasis is properly laid on the importance of dividing the bridge of tissue between the fistula and the bowel as nearly as possible at right angles to the sphincter. It is rather surprising, then, to see in the illustrations, which are admirably drawn, and evidently from life, that the old faulty twist is given to the director, by which the incision is necessarily made oblique. We certainly doubt, also, whether Mr. Allingham would agree with what is depicted on page 87 as "The Proper Method of using Allingham's Scissors and Director." Speaking generally, however, the book is good; it is very readable throughout, and there is an air of common sense about its recommendations as to treatment, which is convincing and satisfactory. The chapters by Mr. Herbert Allingham on cancer of the rectum and on colotomy are very useful. His views on both these subjects are now fairly well known, and they are admirably put in this book. By dint of a series of diagramatic illustrations he throws much light on the anatomical considerations which are involved in colotomy, and on the proceedings which are necessary, if the full benefits of the operation are to be obtained, not merely as a means of relieving obstruction, but as a method for providing a useful artificial anus.

The Composition of Expired Air and its Effects upon Animal Life. By J. S. Billings, M.D., S. Weir-Mitchell, M.D., and D. H. Bergey, M.D. (City of Washington: Published by the Smithsonian Institution.)

Very interesting to the physical investigator who is endowed with a tincture of philosophy, and very important from the standpoint of the hospital administrator, the architect, the churchwarden, the theatre-manager, and other persons who supervise large assemblages of people when shut up temporarily or permanently within four walls, is the latest "Contribution to Knowledge" published by the Smithsonian Institution at Washington City. Expired air—air breathed over and over again by large numbers of living creatures—is unpleasant and unwholesome; in course of time, and when breathed by very large numbers, as in the historical instance of the Black Hole of Calcutta, it becomes poisonous in a deadly degree. Why is this? Science has long wanted to know; and in the persons of many different investigators has made many efforts to find out. One might have thought that inductive science, operating on physical materials, and by physical methods, would have been able to come to some definite conclusion—a conclusion which every competent investigator in every country would be obliged by his own demonstrations of facts, willy nilly, to confirm. By no means. And herein the scientist who is blest with a little philosophy finds occasion for a few moments' indulgence in a little quiet amusement. "It is your mental philosopher, your metaphysician, and your theologian who never can come to any single definite conclusion, but who always divides his forces into opposite and hostile camps," says the man of physical science. "Softly," may now reply the mental philosopher. "Here we have a physical controversy about so simple a thing as the composition of expired air; and this small matter of physical science has already a controversial history a quarter of a century long, already opposing camps, each distinguished by great names, and is at this very moment as far off from solution and definite reconcilement as ever." What is it in expired air which makes it poisonous? An organic alkaloid, an animal poison akin to the ptomaines, answered Brown-Séquard, d'Arsonval, and others nearly a quarter of a century ago. Therefrom has sprung a whole literature which Drs. Billings, Weir-Mitchell, and Bergey have very tersely summarised for the busy reader. What is the constituent in expired air which makes it deadly? has continued to be asked; and those experimenters, among whom our American contemporaries may be named, who differ from Brown-Séquard and his legions, have answered that it is not so much a constituent as the absence of a constituent; it is the lack of oxygen in expired air, combined with the presence of excess of carbonic acid—and not an organic poison at all. This is a summary of the main contentions of the new "Contribution to Knowledge"; but, though a summary, it is by no means an appraisement of its value. The memoir is exceedingly valuable, and well worthy of an extended review did space permit. To the scientific mind it is as interesting as a novel, and to the philosopher it is an excellent corrective and modifier of any pretensions to scientific infallibility. It proves nothing conclusively; but in the fullest sense of the word it is a "contribution to knowledge." In the hands of a practical person who knows how to use it, the memoir may render service in the fields of practical ventilation, and otherwise of very great utility indeed. If we may make a single criticism of an adverse kind, we would suggest that the Smithsonian Institution have very materially diminished the every-day value of the "Contribution" by publishing it in such a cumbrous and unportable form. Let it be summarised, popularised in style, and published in a convenient form, and it will probably be sold in tens of thousands. Perhaps the authors are too "scientific" to desire this? We hope not.

